# Rapid improvement of grain appearance in three-line hybrid rice via CRISPR/Cas9 editing of grain size genes

**DOI:** 10.1007/s00122-024-04627-8

**Published:** 2024-06-27

**Authors:** Juan Huang, Weiwei Chen, Lijun Gao, Dongjin Qing, Yinghua Pan, Weiyong Zhou, Hao Wu, Jingcheng Li, Chonglie Ma, Changlan Zhu, Gaoxing Dai, Guofu Deng

**Affiliations:** 1 Rice Research Institute, Guangxi Academy of Agricultural Sciences/Guangxi Key Laboratory of Rice Genetics and Breeding, Nanning, 530007 People’s Republic of China; 2 Guangxi Academy of Agricultural Sciences/Guangxi Crop Genetic Improvement and Biotechnology Laboratory, Nanning, 530007 People’s Republic of China; 3 Key Laboratory of Crop Physiology, Ecology and Genetic Breeding, Ministry of Education, Jiangxi Agricultural University, Nanchang, 330045 People’s Republic of China

## Abstract

**Key message:**

Genetic editing of grain size genes quickly improves three-line hybrid rice parents to increase the appearance quality and yield of hybrid rice.

**Abstract:**

Grain size affects rice yield and quality. In this study, we used CRISPR/Cas9 to edit the grain size gene *GW8* in the maintainer line WaitaiB (WTB) and restorer line Guanghui998 (GH998). The new slender sterile line WTEA (*gw8*) was obtained in the BC_2_F_1_ generation by transferring the grain mutation of the maintainer plant to the corresponding sterile line WantaiA (WTA, *GW8*) in the T_1_ generation. Two slender restorer lines, GH998E1 (*gw8(II)*) and GH998E2 (*gw8(I)*), were obtained in T_1_ generation. In the early stage, new sterile and restorer lines in grain mutations were created by targeted editing of *GS3*, *TGW3*, and *GW8* genes. These parental lines were mated to detect the impact of grain-type mutations on hybrid rice yield and quality. Mutations in *gs3*, *gw8,* and *tgw3* had a minimal impact on agronomic traits except the grain size and thousand-grain weight. The decrease in grain width in the combination mainly came from *gw8/gw8*, *gs3/gs3* increased the grain length, *gs3/gs3*-*gw8/gw8* had a more significant effect on the grain length, and *gs3/gs3-gw8/gw8(I)* contributed more to grain length than *gs3/gs3-gw8/gw8(II)*. The heterozygous *TGW3/tgw3* may not significantly increase grain length. Electron microscopy revealed that the low-chalky slender-grain variety had a cylindrical grain shape, a uniform distribution of endosperm cells, and tightly arranged starch grains. Quantitative fluorescence analysis of endospermdevelopment-related genes showed that the combination of slender grain hybrid rice caused by *gs3* and *gw8* mutations promoted endosperm development and improved appearance quality. An appropriate grain size mutation resulted in hybrid rice varieties with high yield and quality.

**Supplementary Information:**

The online version contains supplementary material available at 10.1007/s00122-024-04627-8.

## Introduction

Heterosis of hybrid rice varieties can improve rice yield; however, its quality has long been criticized (Wang et al. 2012). Grain size affects not only the rice yield but also its quality( Xu et al. 2004; Fitzgerald et al. 2009). With the increasingly high requirements for rice quality in modern society, the high-end rice market has strict requirements for the appearance of rice, where a slender grain, less chalkiness, and crystal clarity are typical appearance characteristics (Wang 2021); therefore, Simiao-type rice (brown rice length ≥ 6.5 mm, brown rice length-to-width ratio ≥ 3.5, T/GDSMM 001–2019) is widely popular in the market (Qiu et al. 2021). The quality of hybrid rice is determined by both the restoring and sterile lines(Shi and Zhu 1994; Huang et al. 2016). Currently, parent breeding is focused on high yield, strong restoration, high combining ability, and resistance (Ren et al. 2016; Zhang et al. 2017). The phenomenon of high chalkiness and chalky rice frequency has a significant impact on the improvement of the appearance quality of hybrid rice (Min et al. 2015). A strict restoration-preservation relationship exists between the maintainer, sterile, and restoring lines. Traditional breeding processes introduce foreign genes through hybridization, which poses the risk of introducing minor or major restoring genes into the maintainer line, whereas the restoring line poses the risk of reducing its restoration intensity, greatly reducing breeding efficiency (Ren et al. 2016). Therefore, an effective way to improve the appearance quality of three-line hybrid rice is to strengthen the grain size selection of parents by genome editing.

The appearance quality of rice includes brown rice length, brown rice width, ratio of rice length to width, chalky rice frequency, chalkiness degree, and translucency (Yang et al. 2001). Chalkiness, which is the white opaque part of the rice endosperm, is an important factor that affects rice quality and determines its commercial value (Tian et al. 2009; Zhou et al. 2015). Rice chalkiness frequency and degree are positively correlated with brown rice width and thickness, but negatively correlated with brown rice and the ratio of rice length to width (Xu et al. 2004). Therefore, the regulation of grain size has a significant impact on chalkiness (Matsuoka and Ashikari 2007; She et al. 2010; Wang et al. 2012, 2015; Liu et al. 2018a; Zhao et al. 2018). To date, more than 60 grain size-related genes have been cloned(Liu et al. 2018b; Chen et al. 2020). Among these, *GS3* and *TGW3* negatively regulate the grain length (GL) and weight of rice, whereas *GW8* positively regulates the grain width (GW), making them important genes for production(Wang et al. 2015; Fan et al. 2006; Hu et al. 2018; Xia et al. 2018; Ying et al. 2018). The traditional hybrid breeding method has a long cycle and low efficiency, and grain size, a quantitative trait, increases the difficulty of variety selection (Philpot et al. 2006). Genome editing technology can directionally modify target genes, providing opportunities for rice variety improvement (Shan et al. 2013). Following years of practice, remarkable results have been achieved in terms of improving rice yield, quality and resistance (Chen et al. 2020; Hui et al. 2021; Shen et al. 2016; Li et al. 2016; Xu et al. 2020; Usman et al. 2021; Huang et al. 2022). In terms of rice grain size, CRISPR/Cas9 technology has been used to edit grain-type genes such as *GS3, GS9*, *GL3.1*, and *GW8* (Shen et al. 2016; Li et al. 2016; Xu et al. 2020; Zhu et al. 2023), resulting in significantly increased rice plant lines with improved yield and quality(Chen et al. 2020; Xu et al. 2020; Huang et al. 2022). However, in these studies, germplasm resources were mainly created in japonica rice varieties, with only a few being used for indica rice varieties, especially in the cultivation of indica three-line hybrid rice sterile lines(Han et al. 2018; Hui et al. 2021).

Mei1A, WantaiA(WTA), and Guihui582(GH582) are the hybrid rice parents with high combining ability bred by our team, and Guanghui998(GH998) is a high-quality multi-spike restorer widely used in southern rice regions; the high-quality hybrid rice variety Meiyou998, composed of MeiA and GH998, has been popular in the southern rice region for more than 10 years(Liang et al. 2001; Dai et al. 2015; Zhou et al. 2019). However, there is still a large gap between them and Simiao-type rice varieties in grain size requirement, which largely limits the utilization efficiency of these hybrid rice parent lines. In previous experiments, we edited the *GS3* gene in the medium-grain maintainer line Mei1B (*GS3,* GL 9.42 mm) to obtain a long-grain maintainer line Mei2B (*gs3,* GL 10.14 mm) and then introduced this mutation into the corresponding sterile line Mei1A to quickly obtain a long-grain sterile line Mei2A (*gs3*). Compared to Mei1B(Mei1A), the appearance quality of Mei2B (Mei2A) was improved (Huang et al. 2022). We also utilized CRISPR/Cas9 to simultaneously edit the *GS3*, *GW8*, and *TGW3* genes in the restorer line GH582 (*GS3GW8TGW3*, GL 8.61 mm, GW 2.82 mm) with short and wide grains and created a new slender grain restorer line, GH582E (*gs3gw8tgw3*, GL 10.20 mm, GW 2.46 mm), which had better appearance quality than GH582 (Huang et al. 2023).

In this experiment, the *GW8* gene was edited in WTB, the maintainer line corresponding to WTA, and restorer line GH998, to rapidly obtain hybrid rice parents with improved appearance quality through grain mutation. Meanwhile, the improved parents with mutations in *gs3*, *tgw3* and *gw8* obtained from simultaneous gene editing were mated with different hybrid rice combinations to transition from germplasm resource creation to breeding applications. Genotype identification, agronomic traits, and quality analyses of the parents and hybrid rice combinations were performed to study the effect of grain shape mutations on improvements in yield and appearance and to select the ideal hybrid rice combinations according to the breeding objectives.

## Materials and methods

### Test materials

The test materials included the indica medium-grain-length maintainer line Mei1B (GL 9.42 mm), the long-grain mutant maintainer line Mei2B (GL 10.14 mm) obtained by *GS3* gene editing, the wider grain maintainer line WTB and their corresponding sterile lines Mei1A, Mei2A, and WTA, the indica broadly amphibious restorer line GH582 and the new slender-grain restorer line GH582E obtained by multi-gene (*GS3GW8TGW3*) editing, all independently selected by our team(Liang et al. 2001; Dai et al. 2015; Zhou et al. 2019; Huang et al. 2022, 2023). In addition, the restorer line GH998, which is widely used in southern rice-growing regions, was used in these experiments. All materials were grown in isolated greenhouses under conditions similar to those in the field environment.

### Genotypic analysis of grain size and rice quality-related genes in the test materials

The grain size genes *GS3*, *TGW3*, and *GW8,* rice quality-related gelatinization temperature gene *alk*, amylose content gene *Wx,* and chalk gene *Chalk5* were selected to detect the allelic distribution of each gene in the test parents via fluorescent molecular marker detection or sequencing (Hirano et al. 1998; Gao et al. 2003; Mao et al. 2010; Li et al. 2014; Wang et al. 2015; Hu et al. 2018; Ying et al. 2018; Xia et al. 2018).

### Design of grain size-related gene targets for test materials

#### Targets design for the maintainer line WTB and restorer line GH998

*GW8* was chosen as the target gene for the wider grains and aromatic hybrid rice parent WTB. Based on the whole genome sequence of *GW8* (*Os08g0531600*), two targets, GW8-A1 agctggagaacagcggcggcggg and GW8-A2 ccgccggcggcgagctgggagct, were designed in exon 1 to improve the possibility of a target gene knockout(KO). The targets of *GW8* for the restorer line GH998 were designed in exon 3, GW8-B1 agaccggaggcaagctggacagg and GW8-B2 gtcagctccggcgaactccacgg (Huang et al. 2023). Meanwhile, two targets, TGW3-S1 gaatccatgtcccggccgagagg and TGW3-S2 ataatagctactacgatccgtgg were designed in exon 1 and 2 based on the sequence of *TGW3 *(*Os03g0841800*) (In the obtained mutant plants of GH998, editing of the *TGW3* gene did not play a role in the grain mutation).

### Construction of intermediate vectors and transformation of recombinant plasmids

According to the sequence of the target loci, primers with an Eco31I restriction enzyme locus (Table S1, the enzyme cut site is underlined) were synthesized and mixed in equal amounts and then denatured and annealed separately. The total volume was 50 µL, including 5 µL of forward primer (100 µM), 5 µL of reverse primer (100 µM), and 40 µL of H_2_O. The reaction procedure comprised denaturing at 95 °C for 10 min, annealing at 55 °C for 10 min, and cooling at 14 °C for 5 min. The gRNA fragments carrying the Eco31I digestion site were ligated to the empty vectors pBWA and pBWD as follows: a ligation system of 10 µL was configured with 2 µL of gRNA fragment(10 ng/µL), 1.5 µL of empty vector, 0.5 µL of Eco31I enzyme(10 u/µL), 0.5 µL of T4-ligase(350 u/µL), 1 µL of 10 × T4-buffer, and 4.5 µL of H_2_O, which was placed and incubated at 37 °C for 2 h. The two constructed vectors containing the editing element and the binary vector fraction were reconstituted using an enzymatic ligation method to obtain the recombinant plasmids CRISPR-Cas9-GW8 and CRISPR-Cas9-GW8/TGW3 (Fig. S2). The two constructs were introduced into the *Agrobacterium tumefaciens* strain EHA105. *Agrobacterium*-mediated transformation was performed by Biorun Biosciences (Wuhan, China).

### Analysis of target locus mutations and grain variation in positive plants

Positive plants were screened using the primer Hyg-F/Hyg-R, and the test materials were amplified with primers to detect mutations at each locus. Positive seedlings and wild-type (WT) material of the same period were planted, and the seeds were collected at maturity. Ten full seeds from each plant were selected and placed in a Wanshen seed tester to measure their length in comparison with that of the WT seeds.

### Creation of slender-grain maintainer line and sterile line

Seeds of harvested T_0_-generation individual plants were sown in 4 × 10 plots, where each individual plant was numbered individually for detection. The primers Hyg-F/Hyg-R were used to amplify the DNA of each single plant within the T_1_-generation plot, and unmarked Nipponbare was used as the control; those in which amplification of the target bands failed were considered transgene-free mutant plants. In the T_1_ generation, homozygous mutant and transgene-free plants with significantly slender grains were selected and named WTEB for the test crossing with the corresponding sterile line WTA of the WTB material. The seeds of the hybrids and individual plants of the corresponding mutant maintainer line were backcrossed twice to obtain stable grain growth of the sterile material and the corresponding maintainer line.

### Acquisition of new restorer lines with grain size mutations

Individual plants that met the mutation expectations were selected in the T_0_ generation, and the mutation loci were sequenced. The individual plants with homozygous mutated loci were harvested and planted as T_1_-generation plants, which were tested for transgene-harboring status based on each plant with the marker gene hygromycin, and the mutant loci were sequenced to detect homozygous mutations. Individual plants with homozygous mutated loci without marker genes that met the expected targets were selected and identified as new restorer line.

### Mating to obtain hybrid rice combinations with different grain sizes

The sterile lines Mei1A and WTA, as well as their corresponding sterile lines obtained via grain size gene editing, were crossed with the restorer lines GH998 and GH582, as well as with the new restorer lines improved via grain editing to obtain different inbred rice combinations.

### Examination of major agronomic traits

The new maintainer and restorer lines with grain gene mutations and their test-crossed hybrid rice combinations were examined for agronomic traits, including plant height, effective tiller number, panicle length, grain length, grain width, ratio of grain length to width, grain number per panicle, filled grain number per panicle, thousand-grain weight, seed-setting rate, and grain weight per plant. Data were analyzed using GraphPad Prism 8 software (GraphPad Software, California).

#### Analysis of major grain quality traits

Major grain quality traits, including the brown rice length, brown rice width, ratio of rice length to width, head rice rate, amylose content, gel consistency, alkali spreading value, translucency, chalky rice frequency, and chalky degree were examined in the combinations of new maintainer and restorer lines with grain size mutations and corresponding test-crossed hybrid rice and the data were analyzed using GraphPad Prism 8.

#### Scanning electron microscopic analysis of grains from hybrid combinations

Six hybrid rice combinations were selected and compared among the three groups based on grain size and genotypic characteristics. The seeds were harvested at maturity, dried, and milled into fine rice before conducting the experiments. Five pieces of milled rice were randomly selected from each sample, and the middle of the grains was tapped with the back of the blade to break them naturally. The cross sections of the rice grains were firmly attached to a conductive carbon film double-sided tape and placed on an ion sputterer sample platform for approximately 30 s of conductive treatment while being observed and photographed with a scanning electron microscope SU8100 (Hitachi, Japan).

#### Fluorescence quantitative analysis of genes related to endosperm development

Considering the close relationship between rice grain size and endosperm development and quality, 10 genes related to starch synthesis and grain filling were selected. On the 5th stage of young panicle development with seed filling at the 10th and 20th days, quantitative fluorescence analysis was performed to detect the expression of 10 genes at different stages of grain development.

## Results

### Grain size and quality-related genotype identification

The genes *ALK*, *Chalk5*, *Wx*^*b*^, *GW8*, *GS3*, and *TGW3* (Hirano et al. 1998; Gao et al. 2003; Mao et al. 2010; Li et al. 2014; Wang et al. 2015; Ying et al. 2018) were genotyped in four parental lines (Mei1B, WTB, GH582, and GH998) that were unedited using fluorescent molecular markers (Ye et al. 2001; Zhang et al. 2019) or genome sequencing (Table S1, 5). The four parental materials were all *Wx*^*b*^ genotypes with low straight-chain starch content, *ALK* genotyping results suggested that the restorer line GH582 had a low pasting temperature, whereas the remaining parental materials had a high pasting temperature. *Chalk5* genotyping test results indicated that the restorer line GH998 and maintainer line WTB were low-chalk genotypes, wheres the rest were high-chalk, genotypes. *GS3* negatively regulates grain length, and a nonsense mutation in its second exon results in the loss of 178 amino acids at the C-terminus of the *GS3* protein, leading to longer grains (*gs3*) (Fan et al. 2006). *GW8* positively regulates grain width and a loss-of-function mutation in Basmati rice is associated with the formation of slender grains (Wang et al. 2012). Sequence analysis of different grain varieties of *TGW3* suggests that the large-grain allele may be at least partially functionally incapacitated (Ying et al. 2018). The genotype detection results of the three grain genes were as follows: *GW8* genotyping test results identified the maintainer line Mei1B as having slender grains, whereas the other three parents were wide-grain genotypes; four parents were short-grained *TGW3* genotypes; the *GS3* genes of GH998 and WTB were long-grained; and the rest were short-grained genotypes. Detailed typing is shown in Table S2.

### Analysis of target locus mutation in maintainer line transgenic seedlings

Five positive plants were obtained after an exon 1 KO of the *GW8* gene in the maintainer line WTB, where the T_0_ generation was heterozygous, with double-peaked sequencing results and no significant changes ingrain size. The seeds of the T_0_ generation were collected and planted as the T_1_ generation for individual plants and observed at the heading stage, showing slender individual plants isolated from strain F176-14. Hence, the leaves were collected to detect the mutation loci. The sequencing results showed that the individual slender mutant plants exhibited a single-base insertion and G-C transition in exon 1, resulting in the early termination of translation, the production of an unwanted protein, and slender grains(Figs. S2, S5B).

### Identification of the *gw8* homozygous mutant maintainer line WantaiEB (WTEB)

**Fig. 1:**
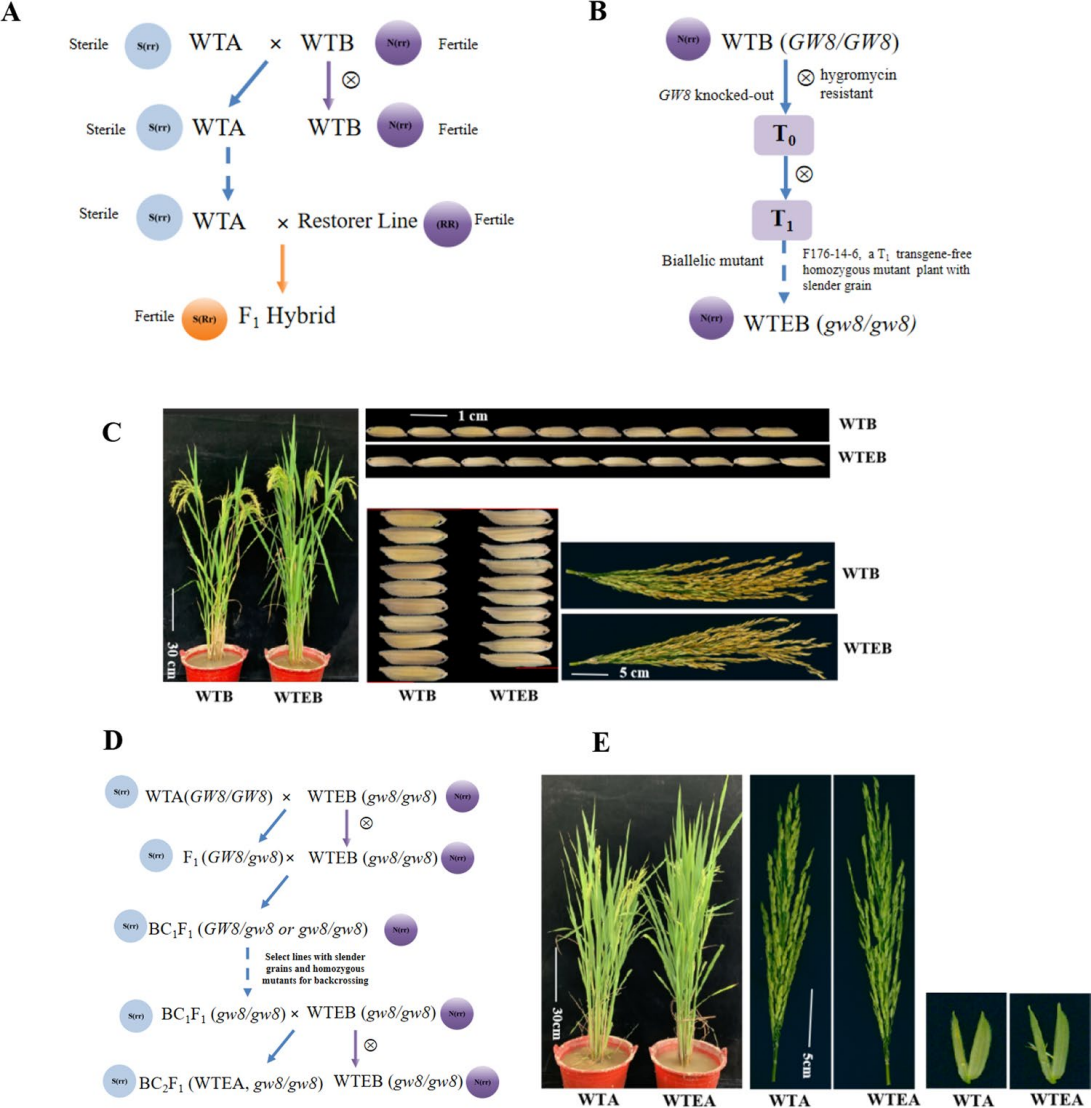
Process for converting the mutant maintainer line WTEB into the mutant male sterile line WTEA. **A** Relationship between WTA and WTB. S represents the cytoplasmic sterile gene, N represents the cytoplasmic normal fertile gene, and r represents the nuclear recessive sterile gene. When the individual genotype was S (rr) (WTA), it was sterile, and the individual N (rr) (WTB) was fertile. When the dominant fertile gene R was in the nucleus of the restorer line, the individuals showed normal fertility regardless of whether the cytoplasm was S or N. The male sterile line WTA (S (rr)) and its corresponding maintainer line WTB (N (rr)) were only different in fertility. Male sterile lines relied on maintainer lines for reproduction and improvement. **B** Breeding process of WTEB. **C** Comparison of the plant, panicle, grain length, and width of WTB and WTEB. **D** Conversion of superior long-grain sterile line WTEA from maintainer line WTEB. E Photographs of the plant, panicle and spikelet of WTA and WTEA. A, B and D were modified according to Huang et al. (2022)

Seeds of the T_0_ generation based on the WTB mutation were harvested and sown using the same specifications as those for the T_1_-generation plants, and the presence or absence of the marker gene in each plant in the plot was tested at the seedling stage using the Hyg-F/ Hyg-R marker(Table S1). An individual plant, F176-14–6, with slender grains, a homozygous mutation, and transgene-free status, was selected from the T_1_-generation mutants of WTB and named WangtaiEB(WTEB) (Fig. 1B).

### Creation of new sterile line WTEA from the maintainer line WTEB

In the homozygous, slender, transgene-free mutant F176-14–6 (WTEB), pollen was collected at the heading stage, tested with WTA, and backcrossed with BC_1_F_1._ Slender grain, homozygous, and transgene-free individual plants were selected from the segregating population and continuously backcrossed with WTEB to BC_2_F_1_ to obtain a new narrow grain sterile line with a homozygous *gw8* mutation and no transgenic component, which was named WantaiEA (WTEA, Fig. 1D, E). The agronomic traits are shown in Table S3.

### Analysis of grain mutations in the restorer line GH998 and selection of new restorer line

**Fig. 2:**
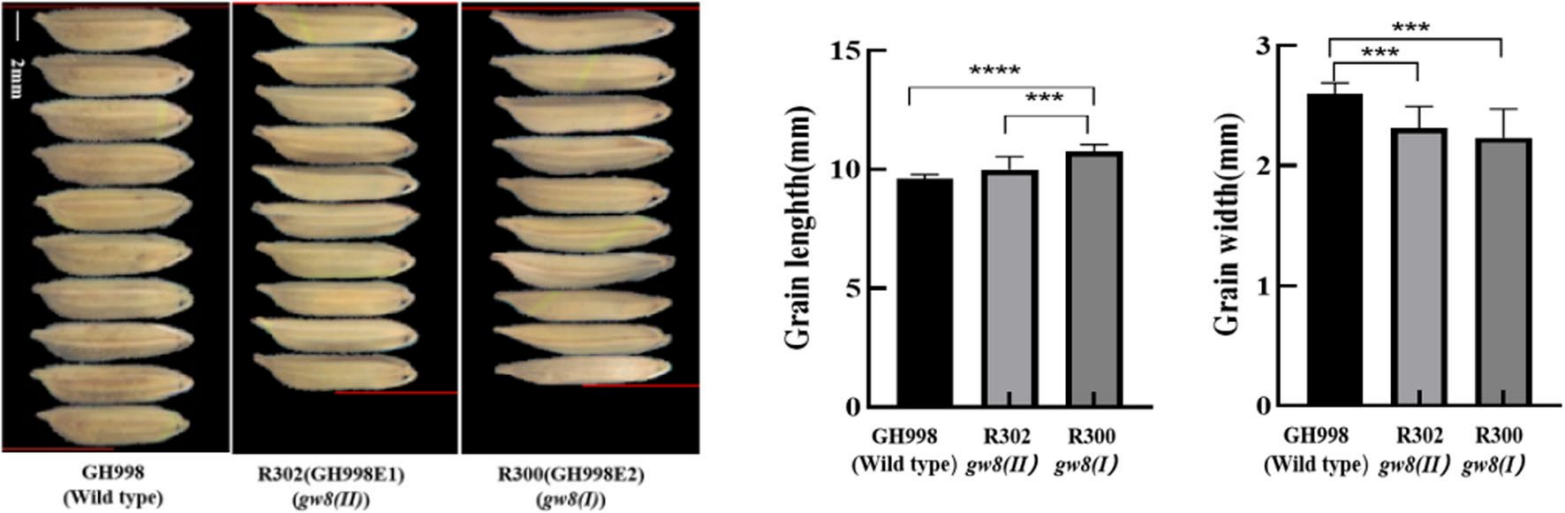
Grain mutations and breeding of new restorer line. ***, ****Indicated the significant difference at *P* levels 0.001 and 0.0001 (*n* = 10)

Twelve T_0_-generation positive plants comprising the test material were obtained by screening with hygromycin gene primers. Among the amplified products, two individual plants, R300 and R302, which had a narrow grain size and were homozygous at the mutation loci, were tested by sequencing. Among them, the individual plant numbered R300 had a base T inserted at target 1, GW8-B1, which led to the premature appearance of a stop codon, forming a truncated protein of only 221 amino acids(Fig. S4 B, C), and the grain size of this plant was significantly slender and longer than that of the WT (Fig. 2). The mutation at target GW8-B2 was invalid and R300 plant did not exhibit mutations at the *TWG3* gene target. The resulting mutant was denoted as *gw8(I)*. R302 had a 1-base A insertion at target GW8 B1, forming a truncated protein of 255 amino acids (Fig. S4A, C). The mutation at target GW8-B2 was also invalid. The *TGW3* gene had a 30 base deletion at the target site in R302, resulting in a reduction of 10 amino acids compared to the WT (Fig. S4 D, E). The corresponding grain size of R302 exhibited a pronounced narrow width; however, its length did not change significantly (Fig. 2), indicating that the absence of 10 amino acids of the *TGW3* site did not affect protein function and remained the short grain genotype (*TGW3*). The resulting mutant was denoted as *gw8(II)*. Based on a comparison of the grain maps, it can be seen that R302 and R300 had significantly reduced grain widths compared with those of the WT GH998. Compared with GH998 in terms of the change in grain length, R302 did not show a significant difference, but R300 exhibited significant grain growth, indicating that in this variety, *gw8(I)* had an increased grain length, but a decreased grain width; *gw8(II)* displayed only a change in the grain width but no change in the grain length (Fig. 2). Therefore, in the R300 and R302 mutants, the *TGW3* gene still remained the short grain genotype (*TGW3*) and did not significantly affect the grain length.

Selected T_0_-generation individual plants, R302 and R300, were grown to obtain T_1_-generation plants, the transgene status of each plant was tested using the hygromycin marker gene, and the mutation loci were sequenced to determine whether a homozygous mutation was present. Individual R302-3 plant, with a transgene-free, homozygous mutation, and narrowed grains, was harvested for grains, and the strain was named GH998E1 (*gw8(II)*). Similarly, the individual plant R300-5 with no marker gene, a homozygous mutation, and narrower and longer grains was harvested and named GH998E2 (*gw8(I)*) (Fig. 2). The agronomic traits are shown in Table S3.

### Breeding applications of sterile and restorer lines with mutations in grain size genes

The long-grain maintainer line Mei2B (*gs3*, Fig. S5A) was obtained from Mei1B by N-terminal functional domain editing of the *GS3* gene, and the *gs3* mutant was transferred to the corresponding sterile line Mei1A (GS3, GL9.42 mm, GW 2.42 mm) to obtain long-grain sterile line Mei2A (*gs3*) (Huang et al. 2022). The new restorer line GH582E (*gs3tgw3gw8(II),* GL10.42 mm, GW 2.42 mm) with a slender grain mutation was created by 3-gene editing in the short, round, and wide grain restorer line GH582(*GS3TGW3GW8*), GL8.61 mm, GW 2.82 mm), and the mutations in the three genes were confirmed by sequencing: *gs3* is a long-grain allelic mutation obtained after editing of the N-terminal functional domain (Fig. S5A); *tgw3* is a long-grain allelic mutation at the exon 1, with a complete knockout of the gene (Fig. S5C); the insertion at the third exon target of *GW8* resulted in a slender allelic mutation of a truncated protein with 255 amino acids (Fig. S5B) (Huang et al. 2023), which is consistent with GH998E2 and was labeled as *gw8 (II)*.

The sterile lines Mei1A (*GS3*) and Mei2A (*gs3*) were test-crossed with the restorer lines GH998 and GH998E2, and the obtained combinations were classified as Group I. Combinations obtained from testing with the restorer lines GH582 (*GS3GW8TGW3*) and GH582E (*gs3tgw3gw8 (II)*) were classified as Group II.

The sterile line WTA and its improved sterile line WTEA were test crossed with the restorer lines GH998, GH998E1, and GH998E2; the combinations obtained were classified as group III, and the combinations obtained were test-crossed with the restorer lines GH582 and GH582E, which were classified as Group IV. The names of the combinations and genotypes of the corresponding grain size genes are listed in Table S4. The main agronomic traits of each combination, such as grain length, grain width, ratio of grain length to width, thousand-grain weight, grain weight per plant, panicle length, seed-setting rate, grain number per panicle, filled grain number per panicle, plant height, and effective tiller number were compared for significance. The hybrid combinations were grown in plots of 4 × 10 in an isolated greenhouse under conditions similar to the field environment.

### Analysis of the main agronomic traits of the mated combinations

**Fig. 3:**
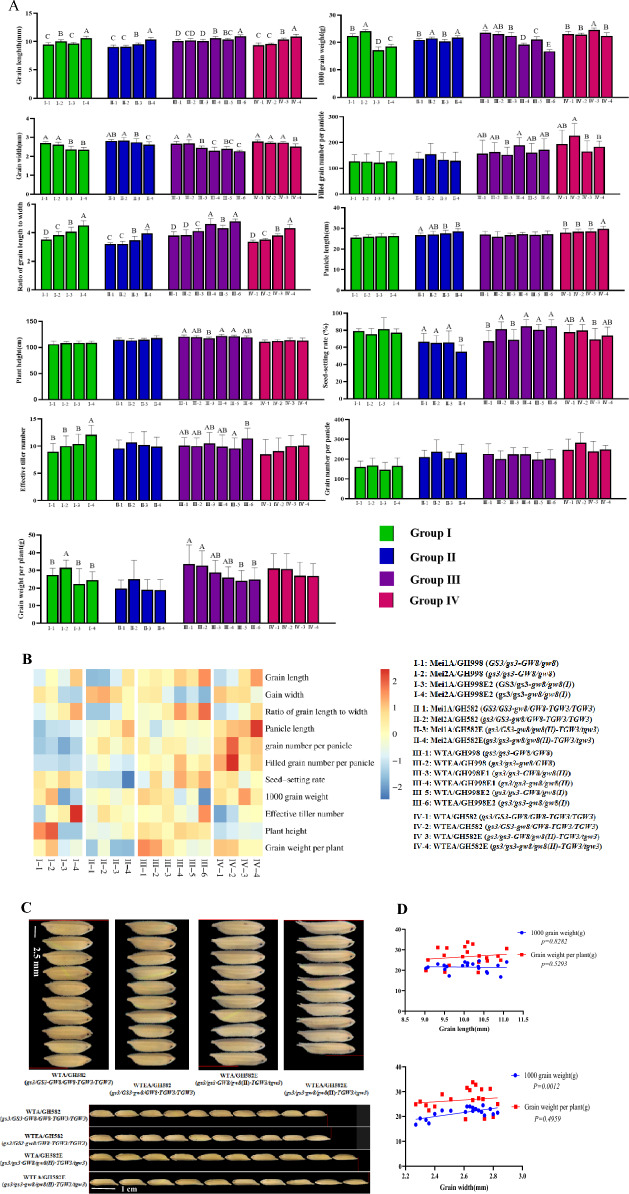
Analysis of agronomic traits of test combinations. **A** Comparison of agronomic traits of tested combinations. **A**, **B** and **C** indicate the significant difference at *P* level 0.05 (*n* = 10). The 16 hybrid combinations were divided into four groups according to the tested parents. The significant differences in major agronomic traits such as grain length, grain width, ratio of grain length to width, thousand-grain weight, grain weight per plant, panicle length, seed-setting rate, grain number per panicle, filled grain number per panicle, plant height, and effective tiller number were compared among the hybrid combinations within the groups. **B** Heat map analysis of agronomic traits in the tested combinations. Heat maps were drawn using PHEATMAP to normalize the agronomic trait data for each combination in four groups, with red and blue indicating high and low values, respectively. **C** Grain size of each combination in Group IV. Comparing the grain length and width of four combinations in Group IV and identifying the corresponding parents and grain genotypes. **D** Correlation analysis between grain size and yield of test combinations. Grain length and width of the 16 hybrid combinations were correlated with thousand-grain weight and grain weight per plant, respectively. A *P*-value less than 0.05 indicates a statistically significant correlation

Mutations affecting grain size are closely related to yield traits (Sakamoto and Matsuoka 2008), and the major agronomic traits of each test-crossed combination were compared to analyze the effects of mutations in the grain size gene on each related trait, especially yield-related traits. From the comparative analysis of agronomic traits among the various combinations in the four groups, the genotypes and grain sizes of the hybrid combinations changed following mutations in the genotype and grain size of the parental material (Fig. 3A, C). Genotype analysis showed that the decrease in the grain width of the hybrid combinations mainly originated from the combination of *gw8/gw8* and, to some extent, in the *gw8/gw8-gs3/gs3* state; *gs3/gs3* increased the length of the grains in the hybrid combinations, and *gs3/gs3-gw8/gw8* had a more significant effect on the increase in grain length (Fig. 3A, Table S4), which is basically consistent with the findings of previous studies (Weng et al. 2008; Wang et al. 2012)*.* Heterozygous *TGW3/tgw3* did not significantly increase the grain length of the hybrid combination (Group II and IV, Fig. 3A, Table S4). *GW8* produced *gw8(I)* and *gw8(II)* mutations in exon 3 and the contribution of *gs3/gs3-gw8/gw8(I)* to grain length was greater than that of *gs3/gs3-gw8/gw8(II)*(Group III, Fig. 3A, Table S4). The mechanism through which *gw8(I)* and *gw8(II)* regulates grain traits needs to be further investigated.

The correlation analysis of grain length and width with the thousand-grain weight and grain weight per plant for each hybrid combination indicated that the correlation between grain length and thousand-grain weight, as well as grain weight per plant, was not significant; the correlation between grain width and grain weight per plant was also not significant, but the correlation with thousand-grain weight was positive and significant (Fig. 3D). From the statistical results, it can be seen that the mutations in grain size genes had a relatively limited effect on panicle length, plant height, grain number per panicle, filled grain number per panicle, and effective tillers in the hybrid combination, but had some effect on the seed-setting rate. In terms of the correlation analysis results, the important factor affecting yield, namely grain weight per plant, was correlated with grain length and width, but none of the correlations were significant. From the comparative analysis of grain weight per plant differences among the major groups, only the grain weights per plant of Groups I and III showed differences between combinations owing to changes in the grain size, but only two classes of differences were observed (Fig. 3A). The heat map analysis indicated that the combinations with reduced grain widths all had an increase in the number of filled grains per panicle, with the increase in the number of filled grains compensating, to some extent, for the influence of the reduced grain width on yield. The effective tillers exhibited a corresponding increase in the combinations with a reduced thousand-grain weight, ensuring the grain weight per plant (Fig. 3B).

### Quality analysis of grain size mutation test-crossed combinations

**Fig. 4:**
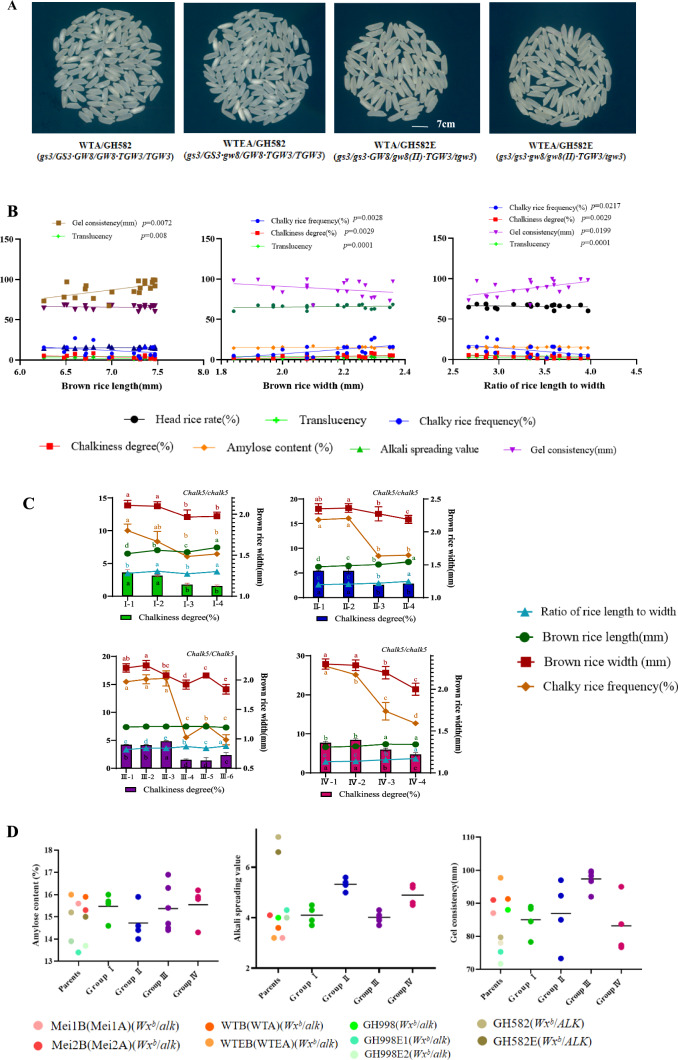
Analysis of rice quality of grain size mutation in combinations. A Brown rice of combinations in Group IV. Comparing the brown rice appearance of four combinations in Group IV and identifying the corresponding parents and grain genotypes. **B** Correlation analysis between grain size and quality of all combinations. Brown rice length and width and the ratio of rice length to width of the 16 hybrid combinations were correlated with head rice rate, chalkiness degree, chalky rice frequency, translucency, gel consistency, alkali spreading, and amylose content, respectively. A *P*-value less than 0.05 indicated a significant correlation. **C** Correspondence analysis of appearance quality and grain width of combinations. Brown rice width was used as vertical axis and chalkiness degree as horizontal axis to draw a trend chart of changes between brown rice length and width, the ratio of rice length to width, chalkiness degree, and chalky rice frequency in four groups. Each group identified the genotype of *Chalk5*. a, b, c, and d indicate the significant difference at *P* level 0.05 (*n* = 10 for brown rice length, brown rice width, and the ratio of rice length to width and *n* = 3 for chalky rice frequency and chalkiness degree). **D** Taste quality analysis of parents and rice combinations. The ranges of gel consistency, amylose content, alkali spreading value of parents, and hybrid combinations are marked with corresponding dots, and the *Wx* and *ALK* genotypes are labeled corresponding to each sample. The parents included Mei1B (Mei1A), Mei2B (Mei2A), WTB (WTA), WTEB (WTEA), GH998, GH998E1, GH998E2, GH582, and GH582E are marked with different colored dots

Quality analysis revealed that combinations with increased rice grain length and decreased width showed significant improvements in appearance quality (Fig. 4A, C). The results of the correlation analysis between grain size and quality traits of the combinations suggested that rice grain length was negatively and significantly correlated with translucency values but positively and significantly correlated with gel consistency. The correlations between grain length and other quality traits were not significant. Grain width was found to be positively correlated with chalkiness and translucency values, but the correlations with other traits were not significant. The correlations between the grain length-to-width ratio and chalky rice frequency, chalkiness, and translucency values were negative, and the correlation with gel consistency was positive and significant; however the correlations with other traits were not significant (Fig. 4B).

Combined with the grain quality-related gene analysis, the grain quality analysis results were consistent with the phenotypes of the corresponding gene types (Figs. 4D, S1). Brown rice length and length-to-width ratio are usually negatively correlated with cooking quality, especially the alkali spreading value (Yang et al. 2001). From the data of the present study, the improvement in appearance quality effected the correlation analysis data for cooking quality; however, this correlation was not significant. The range of data variation in terms of the cooking quality of each parent and the hybrid combination was analyzed in combination with the genotype, and the data indicators were closely related to the functional performance of the related genes; however, the correlation with grain size variations was not significant. In particular, the data of the alkali spreading values of both parents and hybrid combinations were consistent with the function of the *ALK* gene, which regulates the alkali spreading value, and the parents with a low alkali spreading value still obtained hybrid combinations with low alkali spreading values; the parents with a high spreading value obtained hybrid combinations with correspondingly higher spreading values. The gene involved in amylose content, *Wx*, of all parents was tested as *Wx*^*b*^, and the amylose content of varieties with this genotype ranged from 13 to 18% (Hirano et al. 1998). As seen from the test results, the amylose content of both parents and hybrid combinations was within the range of gene function control, indicating the limited effect of grain size on this trait and the dominating role of the *Wx*^*b*^ gene function in this trait.

*The Chalk5* gene affects the chalkiness by influencing the protein accumulation and degradation pathway (Li et al. 2014). The results of the grain size, chalkiness tests, and genotypes of the hybrid combinations showed that the hybrid combinations with highly chalky *Chalk5/Chalk5* (*Chalk5/chalk5*) genotypes and wider grains had high chalkiness, whereas those with slender grains had significantly lower chalky grain frequency and chalkiness. The hybrid combinations with low chalkiness *chalk5/chalk5* genotypes and wider grains had higher chalky rice frequency and chalkiness degree, whereas the combinations with slender grains had lower chalky rice frequency and chalkiness degree (Fig. 4C). These phenomena indicate that when genes conferring wide grains and a highly chalky phenotype coexist, the grains have high chalkiness; however, in slender grains, the effect of genes conferring a highly chalky phenotype on chalkiness is weak, and the effect of grain width on chalkiness is higher than that of chalky genes.

### Scanning electron microscopic analysis of endosperm in hybrid combination

**Fig. 5:**
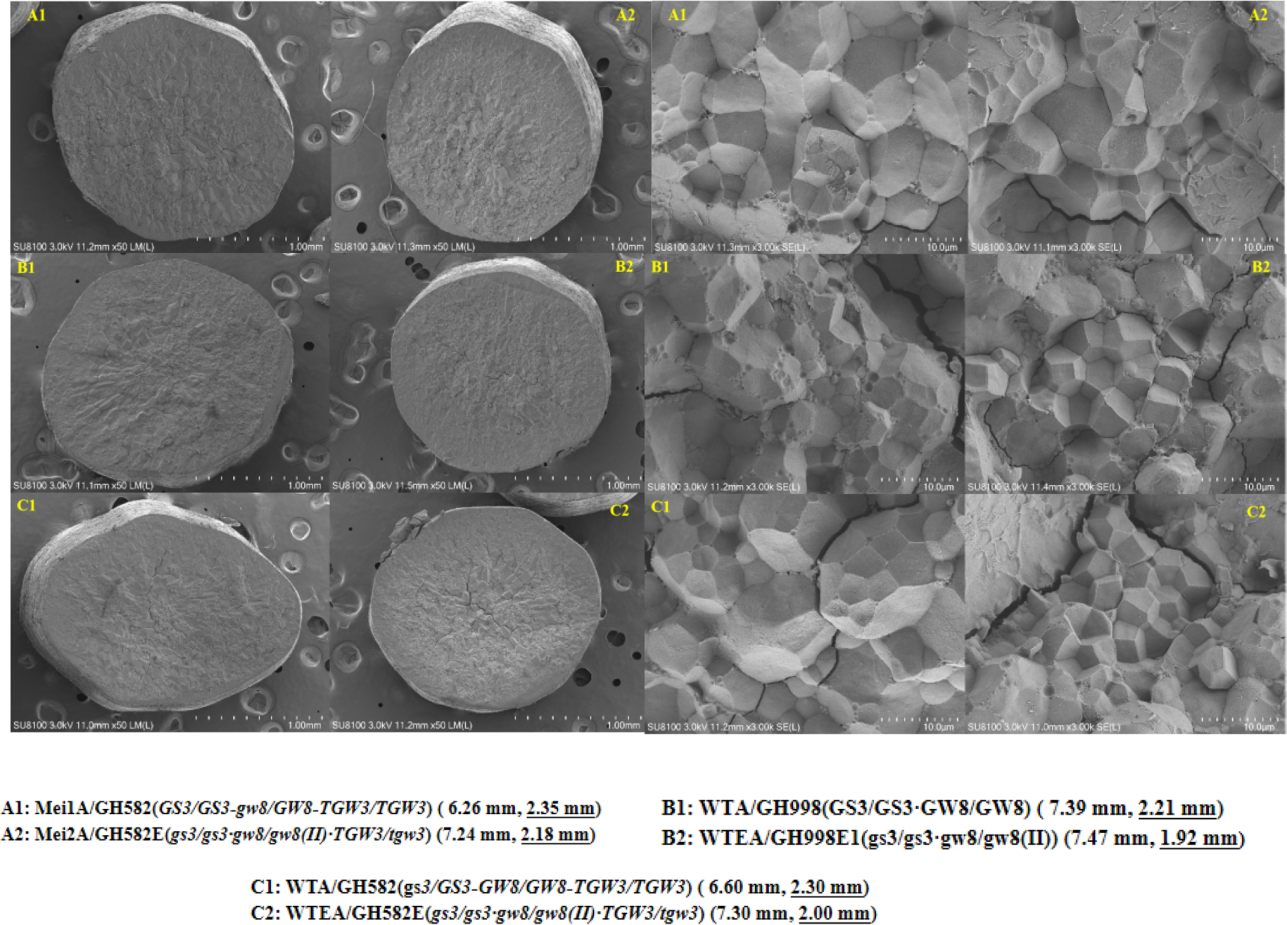
Electron microscopic analyses of transversely fractured mid-region of the test combinations. Left, overall cross-section of brown rice under electron microscopy scanning. Right, arrangement of starch grains in the middle of the rice grains. A1, B1, and C1 hybrid samples, tested by wide type parents, all had wider grains, whereas A2, B2, and C2, obtained by mating the parents with grain mutation, had significantly lower grain widths

Based on the cross-sectional morphology, A1, B1, and C1 showed elliptical or flattened shapes owing to their wider grains, especially for the C1 material. The endosperm cells, based on the cross sections of the three samples, showed an obvious radial shape and were not evenly arranged over the entire cross-section, showing a denser abdomen and decreased distribution on the back. A2, B2, and C2 showed cylindrical shapes under the scanning electron microscope owing to narrower grains, and the starch grains based on the cross sections were more evenly distributed without clear ventral–dorsal distinctions than A1, B1, and C1. From the analysis of the morphology and arrangement of each combination of starch grains, the starch grains of A1, B1, and C1 were mostly spherical with blunt angles, different grain sizes, and a loose arrangement compared to those of A2, B2, and C2. The starch grains of A2, B2, and C2 had a regular polyhedral crystalline shape and were closely arranged. In partiular, multiple starch grains of CB2 and C2 were closely arranged into seamless composite starch granules, indicating that starch was well developed in these two samples. Combined with the results of the rice quality analysis, increasing the length of rice grains and their length-to-width ratio, or decreasing their width could improve the distribution and arrangement of starch grains in rice grains, reduce chalkiness, and improve grain quality. The slender variety with low chalkiness had a cylindrical grain shape, uniform distribution of endosperm cells, and a tight arrangement of starch grains, indicating that the change in grain shape might have caused a change in this pathway during seed filling (Fig. 5).

#### Quantitative fluorescence analysis of genes related to endosperm development in different hybrid combination grains

**Fig. 6:**
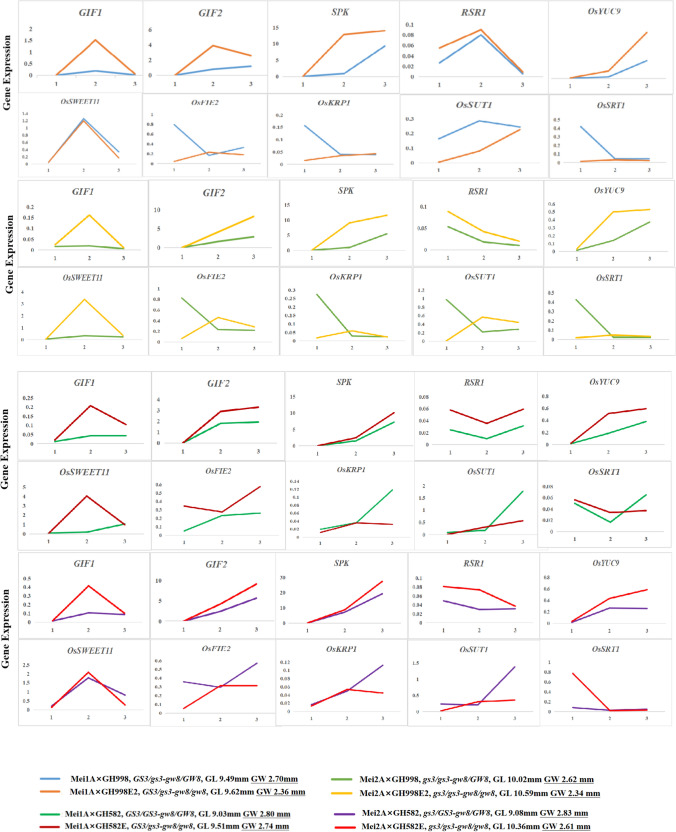
Comparison of gene expression related to endosperm development at different stages of grain development in hybrid combinations. Quantitative fluorescence analysis of 10 endosperm development-related genes. The relative expression of genes was calculated using the 2-ΔCT method with the *Actin* gene as an internal reference. The measurements were repeated three times and the average value was plotted on a line graph. 1: Fifth stage of young panicle development (17 cm). 2: Seed filling on the 10th day. 3: Seed filling on the 20th day

At three different periods of rice development ( the fifth stage of young panicle development, seed filling on the 10th day, and seed filling on the 20th day), quantitative fluorescence analysis was conducted on the spikelets and grains of the hybrid combinations to detect the expression of 10 genes related to grain development (Zhang et al. 2021). At the most critical stage of grain formation (seed filling on the 10th day), the expression levels of endosperm development-related genes in slender grain combinations were usually higher than those in wide grain combinations, indicating that slender grain combinations may improve rice quality by affecting embryo development (Fig. 6).

**Fig. 7:**
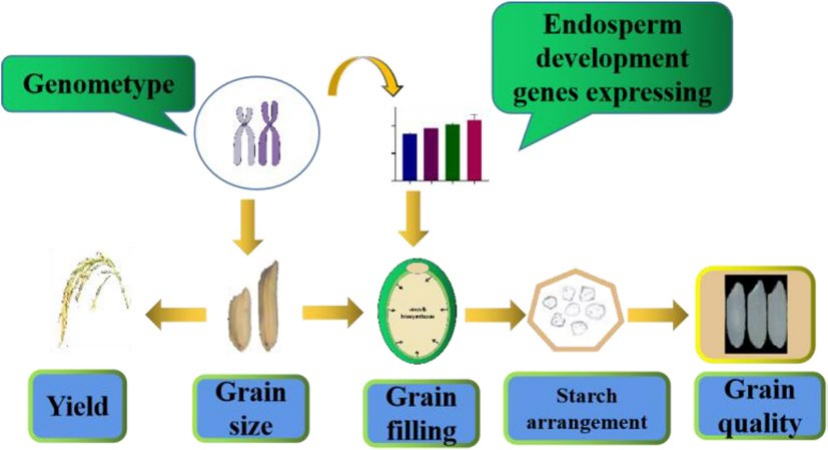
Schematic diagram of grain size and quality formation

Analysis of grain size, genotype, quality, and endosperm development-related genes showed that grain size genes of the hybrid combinations determined the grain shape and affected the expression of endosperm development genes. Grain size and endosperm development genes jointly affected the development of grain endosperm, thereby affecting the final appearance quality of the grains (Fig. 7).

## Discussion

### Gene editing quickly creates excellent hybrid rice parents

Conventional rice breeding mainly relies on genetic recombination and mutation to create new varieties after multiple selections, which is time-consuming and labor-intensive. In molecular marker-assisted breeding, marker loss can occur due to multi-generation backcrossing and chain accumulation, which affects the crop breeding process (Wang et al. 2018). As a quantitative trait, grain size is affected by the growth period and cultivation environment, which increases the difficulty of grain selection (Philpot et al. 2006). The successful matching of the three-line hybrid rice has significantly contributed to China’s food security (Yuan and Tang 1999). At present, three-line hybrid rice breeding still faces huge challenges such as low efficiency in breeding superior retainer lines and the high cost of seed production. In particular, the introduction of desirable exogenous genes into improved retainer and sterile lines using traditional cross-selection methods is accompanied by the risk of infiltration of micro or master recovery genes, which significantly reduces the efficiency of hybrid rice breeding (Ren et al. 2016). CRISPR-Cas9 gene editing technology can be used to efficiently and precisely acquire desired traits, mitigate the limitations of traditional breeding methods, and created a new era of crop improvement (Haque et al. 2018; Mishra et al. 2018). Several experiments on grain size gene editing have demonstrated that rice materials with multiple grain size mutations can be obtained using CRISPR-Cas9 based on the characteristics of different grain size genes (Chen et al. 2020; Xu et al. 2015; Shen et al. 2016; Li et al. 2016; Shen et al. 2017). In this experiment, CRISPR-Cas9 was applied to improve the maintainer lines and transfer the corresponding sterile lines, and the sterile lines with the target trait mutations were obtained in only approximately 2 years. Moreover, the restorer lines with the target trait mutations were bred and ready for application in the mating of new varieties in only one year, which greatly facilitated the breeding process of new hybrid rice varieties compared to the conventional selection process of three-line sterile lines. Using gene editing technology to mutate the endogenous grain size genes of these hybrid rice parents, traits that meet improvement targets can be obtained without the introduction of exogenous genes via hybridization and other means. This not only shortens the breeding time but also avoids the risk of changes in the restoration and maintenance of relationships brought about by the introduction of exogenous genes, promoting the utilization value of hybrid rice parents and the effective use of existing resources.

### Genetic editing of grain size genes significantly improves the appearance quality of hybrid rice

Chalkiness, the white, opaque part of the rice endosperm, is an important factor affecting grain quality (Fujita et al. 2007), and its formation is controlled by multiple genes (Kang et al. 2005; Wang et al. 2008; She et al. 2010). In complex regulatory pathways, grain size has an undeniable impact on chalkiness formation. Through grain size gene editing, the grain length of the hybrid rice parents increased, the grains became slender, the translucency increased, and the chalky rice frequency and chalkiness degree decreased (i.e., the appearance quality was significantly improved). The results of genotype testing and rice quality analysis showed that regardless of whether the hybrid rice combination harbored a high (*Chalk5/Chalk5* or *Chalk5/chalk5*) or a low (*chalk5/chalk5*) chalkiness genotype, the wide grain combination maintained a high chalkiness degree and chalky rice frequency, whereas the slender grain combination was not affected by the high chalkiness genotype, and the chalkiness decreased. Thus, changing the grain length and width can efficiently improve the quality of the appearance of the variety. Scanning electron microscopy results indicated that slender varieties with low chalkiness had uniform endosperm cell distribution and closely arranged starch grains. Slender varieties cause the entire grain size to change from a flat shape in wide grains to a cylindrical shape. The change in grain size suggests that thin and long cylindrical grains facilitate a uniform distribution of nutrients in the endosperm during the filling period, thus reducing the occurrence of chalk (Gu et al. 2001). Quantitative fluorescence analysis of genes related to endosperm development in hybrid combinations showed that the expression levels of genes promoting endosperm development in slender-grain combinations were higher than those in wide-grain combinations (Fig. 6). These phenomena suggest that grain size genes while changing the length and width of the grain, influence the distribution of nutrients and the filling of starch granules inside the whole grain (Fig. 7); however, the associated pathway requires further verification.

### Mutation in grain size balances yield and quality

Placing special emphasis on a slender grain shape affects the thousand-grain weight and individual plant weight, resulting in a low yield of high-quality rice varieties (Jun 1985). In the comprehensive comparison of the combinations in all groups, especially II and V, there was no significant difference in single plant grain weights and no obvious effects on yield, despite variations in grain size, but the quality of appearance improved with increasing grain length-to-width ratio. Combined with the analysis of grain size gene effects, the decrease in grain width in the hybrid combinations was mainly attributed to the *gw8/gw8* combination; *gs3/gs3* increased the grain length; *gs3/gs3-gw8/gw8* had a more evident effect on increases in the grain length, and *gs3/gs3-gw8/gw8(I)* contributed more to grain length than *gs3/gs3-gw8/gw8*. Functional regulation of grain size genes is important for obtaining desirable grain size mutations. Using gene editing technology and molecular marker detection to select suitable parental materials and target genes, three-line hybrid rice varieties with both improved quality and yield can be obtained with an increase in their breeding efficiency.

### Supplementary Information

Below is the link to the electronic supplementary material.

Supplementary file1 (DOCX 916 kb)

## References

[CR58] Chen Y, Zhu A, Xue P, Wen X, Cao Y, Wang B, Zhang Y, Shah L, Cheng S, Cao L, Zhang Y (2020). Efects of GS3 and GL3.1 for grain size editing by CRISPR/Cas9 in rice. Rice Sci.

[CR1] Dai G, Deng G, Chen R, Liang S, Zhou W, Gao L, Zhang Z (2015). Breeding and application of a new two-line super rice variety Guiliangyou 2 for early/late-cropping dual-usage. J South Agric.

[CR2] Fan C, Xing Y, Mao H, Lu T, Han B, Xu C, Li X, Zhang Q (2006). GS3, a major QTL for grain length and weight and minor QTL for grain width and thickness in rice, encodes a putative transmembrane protein. Theor Appl Genet.

[CR3] Fitzgerald M, McCouch SR, Hall RD (2009). Not just a grain of rice: the quest for quality. Trends Plant Sci.

[CR4] Fujita N, Yoshida M, Kondo T, Saito K, Utsumi Y, Tokunaga T, Nishi A, Satoh H, Park J, Jane J, Miyao A, Hirochika H, Nakamura Y (2007). Characterization of SSIIIa-deficient mutants of rice: The function of SSIIIa and pleiotropic effects by SSIIIa deficiency in the rice endosperm. Plant Physiol.

[CR5] Gao Z, Zeng D, Gui X, Zhou Y, Yan M, Huang D, Li J, Qian Q (2003). Map-based cloning of the ALK gene, which controls the gelatinization temperature of rice. Sci China Ser C Life Sci.

[CR6] Gu Y, Xiong F, Wang Z, Chen G, Li W (2001). A contrast of the endosperm development between rice and wheat. J Nanjing Normal Univ.

[CR7] Han Y, Luo D, Usman B, Nawaz G, Zhao N, Liu F, Li R (2018) Development of high yielding glutinous cytoplasmic male sterile rice (*Oryza sativa* L.) lines through CRISPR/Cas9 based mutagenesis of *Wx* and *TGW6* and proteomic analysis of anther. Agronomy 8(12):290

[CR8] Haque E, Taniguchi H, Hassan M, Bhowmik P, Karim M, Smiech M, Zhao K, Rahman M, Islam T (2018). Application of CRISPR/Cas9 genome editing technology for the improvement of crops cultivated in tropical climates: Recent progress, prospects, and challenges. Front Plant Sci.

[CR9] Hirano H, Eiguchi M, Sano Y (1998). A single base change altered the regulation of the waxy gene at the posttranscriptional level during the domestication of rice. Mol Biol Evol.

[CR10] Hu Z, Lu S, Wang M, He H, Sun L, Wang H, Liu X, Jiang L, Sun J, Xin X, Kong W, Chu C, Xue H, Yang J, Luo X, Liu J (2018). A novel QTL *qTGW3* encodes the GSK3/SHAGGY-like Kinase OsGSK5/OsSK41 that interacts with OsARF4 to negatively regulate grain size and weight in rice. Mol Plant.

[CR13] Huang Z, Lv Q, Xin Y, Fu X, Peng Y, Yuan L (2016). Study on quality traits and inheritance of hybrid rice and its parents. China Rice.

[CR11] Huang J, Gao L, Luo S, Liu K, Qin D, Pan Y, Dai G, Deng G, Zhu C (2022). The genetic editing of *GS3* via CRISPR/Cas9 accelerates the breeding of three-line hybrid rice with superior yield and grain quality. Mol Breed.

[CR12] Huang J, Gao L, Li J, Zhou W, Deng G, Pan Y, Qing D, Wu H, Dai G (2023). Improving restorer line appearance quality of three-line hybrid rice via CRISPR/Cas9 technology. Southwest China J Agri Sci.

[CR14] Hui S, Li H, Mawia A, Zhou L, Cai J, Ahmad S, Lai C, Wang J, Jiao G, Xie L, Shao G, Sheng Z, Tang S, Wang J, Wei X, Hu S, Hu P (2021) Production of aromatic three-line hybrid rice using novel alleles of *BADH2*. Plant Biotechnol J 59–74 10.1111/pbi.13695 PMC8710899 34465003

[CR15] Jun BT (1985). Studies on inheritance of grain size and shape in rice. Crops.

[CR16] Kang H, Park S, Matsuoka M, An G (2005). White-core endosperm floury endosperm-4in rice is generated by knockout mutations in the C-type pyruvate orthophosphate dikinase gene (*OsPPDKB*). Plant J.

[CR18] Li Y, Fan C, Xing Y, Yun P, Luo L, Yan B, Peng B, Xie W, Wang G, Li X, Xiao J, Xu C, He Y (2014). *Chalk5* encodes a vacuolar H+-translocating pyrophosphatase influencing grain chalkiness in rice. Nat Genet.

[CR17] Li M, Li X, Zhou Z, Wu P, Fang M, Pan X, Lin Q, Luo W, Wu G, Li H (2016). Reassessment of the four yield-related genes *Gn1a*, *DEP1*, *GS3*, and *IPA1* in rice using a CRISPR/Cas9 system. Front Plant Sci.

[CR19] Liang S, Zhou M, Deng G, Chen R, Wu M (2001). Breeding of indica CMS line MeiA and its characters analysis. Guangxi Agric Sci.

[CR20] Liu Q, Han R, Wu K, Zhang J, Ye Y, Wang S, Chen J, Pan Y, Li Q, Xu X, Zhou J, Tao D, Wu Y, Fu X (2018). G-protein βγ subunits determine grain size through interaction with MADS-domain transcription factors in rice. Nat Commun.

[CR21] Liu X, Mu C, Zhou C, Cheng Z, Jiang L, Wang J (2018). Research progress on cloning and regulation mechanism of rice grain shape Genes. Chin J Rice Sci.

[CR22] Mao H, Sun S, Yao J, Wang C, Yu S, Xu C, Li X, Zhang Q (2010). Linking differential domain functions of the GS3 protein to natural variation of grain size in rice. PNAS.

[CR23] Matsuoka M, Ashikari M (2007). A quantitative trait locus regulating rice grain width. Nat Genet.

[CR24] Min J, Zhu Z, Sun C, Zhang L, Tang S (2015). Analysis of grain quality of major Indica hybrid rice varieties in China. Hybrid Rice.

[CR25] Mishra R, Joshi R, Zhao K (2018). Genome editing in rice: recent advances, challenges, and future implications. Front Plant Sci.

[CR26] Philpot K, Martin M, JR V, Willoughby D, Fitzgerald A, (2006). Environmental factors that affect the ability of amylose to contribute to retrogradation in gels made from rice flour. J Agric Food Chem.

[CR27] Qiu Z, Tan Y, Lan J, Sheng G, He H, Luo L, Li Y (2021). Yexiangyou Lisi, a new high-quality late hybrid Rice combination. Hybrid Rice.

[CR28] Ren G, Yan L, Xie H (2016). Retrospective and perspective on indica three-line hybrid rice breeding research in China. Chin Sci Bull.

[CR29] Sakamoto T, Matsuoka M (2008). Identifying and exploiting grain yield genes in rice. Curr Opin Plant Biol.

[CR30] Shan Q, Wang Y, Li J, Zhang Y, Chen K, Liang K, Liu J, Jeff J, Qiu J, Gao C (2013). Targeted genome modification of crop plants using a CRISPR-Cas system. Nat Biotechnol.

[CR31] She K, Kusano H, Koizumi K, Yamakawa H, Hakata M, Imamura T, Fukuda M, Naito N, Tsurumaki Y, Yaeshima M, Tsuge T, Matsumoto K, Kudoh M, Itoh E, Kikuchi S, Kishimoto N, Yazaki J, Ando T, Yano M, Aoyama T, Sasaki T, Satoh H, Shimada H (2010). A novel factor *FLOURY ENDOSPERM2* is involved in regulation of rice grain size and starch quality. Plant Cell.

[CR33] Shen L, Wang C, Fu Y, Wang J, Liu Q, Zhang X, Yan C, Qian Q, Wang K (2016). QTL editing confers opposing yield performance in different rice varieties. J Integr Plant Biol.

[CR32] Shen L, Li J, Fu Y, Wang J, Hua Y, Jiao X, Yan C, Wang K (2017). Orientation improvement of grain length and grain number in rice by using CRISPR/Cas9 System. Chin J Rice Sci.

[CR34] Shi C, Zhu J (1994). Analysis of seed and maternal effects for character of cooking quality in Indica rice. Chinese J Rice Sci.

[CR35] Tian Z, Qian Q, Liu Q, Yan M, Liu X, Yan C, Liu G, Gao Z, Tang S, Zeng D, Wang Y, Yu J, Gu M, Li J (2009). Allelic diversities in rice starch biosynthesis lead to a diverse array of rice eating and cooking qualities. PNAS.

[CR36] Usman B, Zhao N, Nawaz G, Qin B, Liu F, Liu Y, Li R (2021). CRISPR/Cas9 guided mutagenesis of *Grain Size 3* confers increased rice (*Oryza sativa* L.) grain length by regulating cysteine proteinase inhibitor and ubiquitin-related proteins. Int J Mol Sci.

[CR37] Wang E, Wang J, Zhu X, Hao W, Wang L, Li Q, Zhang L, Li Q, Zhang L, He W, Lu B, Lin H, Ma H, Zhang G, He Z (2008). Control of rice grain-filling and yield by a gene with a potential signature of domestication. Nat Genet.

[CR38] Wang F (2021). Achievements and prospects of hybrid rice breeding—review of 50 years’ research on hybrid rice by rice research institute of Guangdong Academy of Agricultural Sciences. Guangdong Agricul Sci.

[CR40] Wang S, Wu K, Yuan Q, Liu X, Liu Z, Lin X, Zeng R, Zhu H, Dong G, Qian Q, Zhang G, Fu X (2012). Control of grain size, shape and quality by *OsSPL16* in rice. Nat Genet.

[CR39] Wang S, Li S, Liu Q, Wu K, Zhang J, Wang S, Wang Y, Chen X, Zhang Y, Gao C, Wang F, Huang H, Fu X (2015). The *OsSPL16-GW7* regulatory module determines grain shape and simultaneously improves rice yield and grain quality. Nat Genet.

[CR41] Wang Z, Zeng D, Qin R, Liu J, Shi C (2018). A novel and pleiotropic factor *SLENDER GRAIN3* is involved in regulating grain size in rice. Rice Sci.

[CR42] Weng J, Wan X, Gao H, Guo T, Su N, Lei C, Zhang X, Cheng Z (2008). Isolation and initial characterization of *GW5*, a major QTL associated with rice grain width and weight. Cell Res.

[CR43] Xia D, Zhou H, Liu R, Dan W, Li P, Wu B, Chen J, Wang L, Gao G, Zhang Q, He Y (2018) *GL3.3*, a novel QTL encoding a GSK3/SHAGGY-like Kinase, epistatically interacts with *GS3* to produce extra-long grains in rice. Mol Plant 11(5):754–756 10.1016/j.molp.2018.03.006 29567448

[CR44] Xu S, Zheng H, Liu L, Pu Q, Li X, Zou D (2020). Improvement of grain shape and fragrance by using CRISPR/Cas9 system. Chin J Rice Sci.

[CR45] Xu Z, Chen W, Ma D, Lu Y, Zhou S, Liu L (2004). Correlations between rice grain shapes and main qualitative characteristics. Acta Agron Sin.

[CR59] Xu R, Li H, Qin R, Li J, Qiu C, Yang Y, Ma H, Li L, Wei P, Yang J (2015). Generation of inheritable and “transgene clean” targeted genome-modifed rice in later generations using the CRISPR/Cas9 system. Sci Rep.

[CR46] Yang L, Bai Y, Zhang P, Xu C, Hu X, Wang W, She D, Chen G (2001). Studies on the correlation between grain shape and grain quality in rice. Hybrid Rice.

[CR47] Ye S, Dhillon S, Ke X, Collins A, Inm D (2001). An efficient procedure for genotyping single nucleotide polymorphisms. Nucleic Acids Res.

[CR48] Ying J, Ma M, Bai C, Huang X, Liu J, Fan Y, Song X (2018). *TGW3*, a major QTL that negatively modulates grain length and weight in rice. Mol Plant.

[CR49] Yuan L, Tang C (1999). Retrospective and current and perspective on hybrid rice breeding. China Rice.

[CR52] Zhang X, Li J, Tang Y, Wen M, Xiao R, Yao X (2017). Breeding and utilization of restorer line Yuhui 2103 with good grain quality in rice. Hybrid Rice.

[CR50] Zhang B, Zhao N, Liu Y, Jia L, Fu Y, He X, Liu K, Xu Z, Bao B (2019). Novel molecular markers for high-throughput sex characterization of Cynoglossus semilaevis. Aquaculture.

[CR51] Zhang J, Niu B, E Z, Chen Z, (2021). Towards understanding the genetic regulations of endosperm development in rice. Chin J Rice Sci.

[CR53] Zhao D, Li Q, Zhang C, Zhang C, Yang Q, Pan L, Ren X, Lu J, Gu M, Liu Q (2018) *GS9* acts as a transcriptional activator to regulate rice grain shape and appearance quality. Nat Commun 9(1) 10.1038/s41467-018-03616-y PMC5869696 29588443

[CR55] Zhou L, Liang S, Ponce K, Marundon S, Ye G, Zhao X (2015). Factors affecting head rice yield and chalkiness in indica rice. Field Crop Res.

[CR56] Zhou W, Chen W, Dai G, Liang H, Zhou M, Chen R, Deng G (2019). Wantaiyou 3158, a new super hybrid rice combination for both early and late season. Hybrid Rice.

[CR57] Zhu X, Gou Y, Heng Y, Ding W, Li Y, Zhou D, Li X, Liang C, Wu C, Wang H, Shen R (2023). Targeted manipulation of grain shape genes effectively improves outcrossing rate and hybrid seed production in rice. Plant Biotechnol J.

