# Supplementary material for: Rapid improvement of grain appearance in three-line hybrid rice via CRISPR/Cas9 editing of grain size genes
Source: Theor Appl Genet. 2024 Jun 27;137(7):173. doi: 10.1007/s00122-024-04627-8 (PMC11211133; doi:10.1007/s00122-024-04627-8)
Supplement: Supplementary file 1 — Supplementary file1 (DOCX 916 kb) [file 122_2024_4627_MOESM1_ESM.docx]

**Juan Huang^1^**^†^ **, Weiwei Chen^1^**^†^ **, Lijun Gao^2^, Dongjin Qing^1^, Yinghua Pan^1^, Weiyong Zhou^1^, Hao Wu^2^, Jingcheng Li^1^, Chonglie Ma^2^, Changlan zhu^3^*****, Gaoxing Dai^1^*** **and Guofu Deng^1^***

1. Rice Research Institute, Guangxi Academy of Agricultural Sciences/Guangxi Key Laboratory of Rice Genetics and Breeding, Nanning, China
2. Guangxi Academy of Agricultural Sciences/Guangxi Crop Genetic Improvement and Biotechnology Laboratory, Nanning, China
3. Key Laboratory of Crop Physiology, Ecology and Genetic Breeding, Ministry of Education, Jiangxi Agricultural University, Nanchang, China

**Genetic editing of grain size genes via CRISPR/Cas9 revealing the mechanism to quickly improve the appearance quality of three-line hybrid rice**

SUPPLEMENTARY INFORMATION


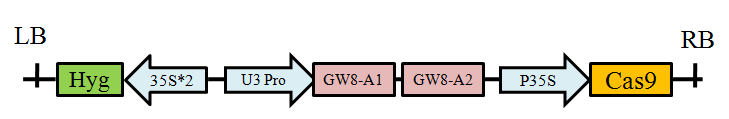


**A**

**B**


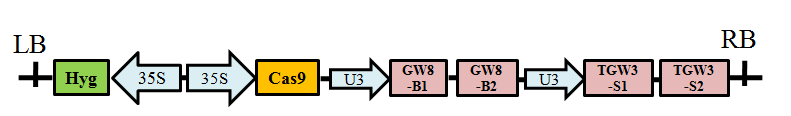


**Fig. S1 Schematic map of the recombinant plasmid**

Note:  **A** Schematic map of the recombinant plasmid CRISPR-Cas9 -GW8 for exon 1 in WTB. **B** Schematic map of the recombinant plasmid CRISPR-Cas9 -GW8/TGW3 in GH998. The inserted fragment region includes knockout targets GW8-A1, GW8-A2 and GW8-B1, GW8-B2, TGW3-S1 and TGW3-S2 activated by U3 promoter, *Cas9* gene activated by ubiquitin promoter and hygromycin phosphotransferase gene activated by 35S promoter. LB, T-DNA left border sequence; RB, T-DNA right border sequence.


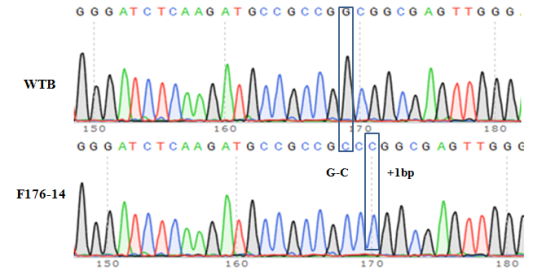


**A**

**B**


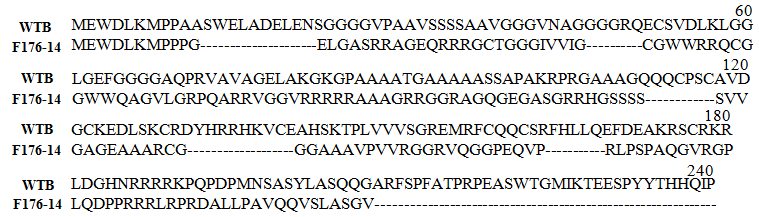


**Fig. S2 Sequencing and amino acid analyses of the homozygous edited transgenic lines of *GW8* in GH998.**

Note: **A** sequencing analysis of the slender transgenic plant and the control of maintainer line WTB.The blue box indicates the mutated base. **B** *GW8* amino acid alignment of the mutant and the control WTB. WTB: wild type maintainer line containing the *GW8* allele; F176-4: mutant maintainer lines containing the edited *gw8* allele.


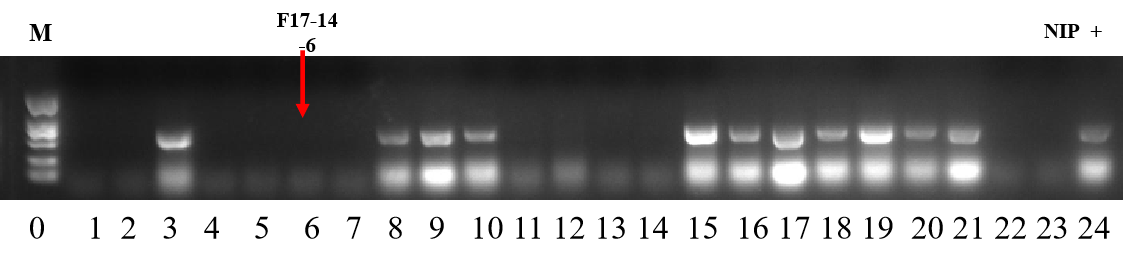


**Fig. S3 PCR identification of the transgene-free transgenic plants**

Note: PCR identification of the WTEB transgene-free transgenic plants. A primer pair Hyg-F/ Hyg-R was used to amplify a fragment of HPT (hygromin phospho transferase) gene. Lane 0: M, Marker 2000. Lane 1-22, individual seedlings of F176-14 mutant; Lane23, NIP, Negative control(Nipponbare); Lane, 24, +, positive control of transgenic line; Lanes with amplified PCR fragment indicated transgene positive. Lanes without amplified PCR fragment indicated transgene-free. Homozygous transgene-free mutant F176-14-6 was pointed with a red arrow.


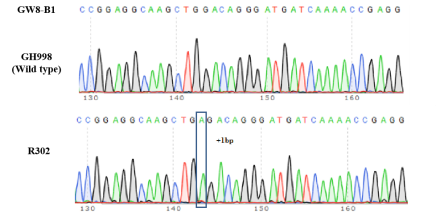

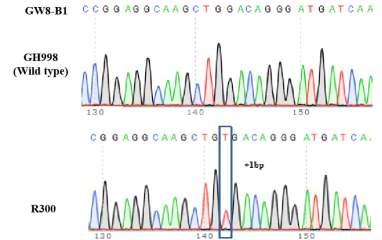


**B**

**A**


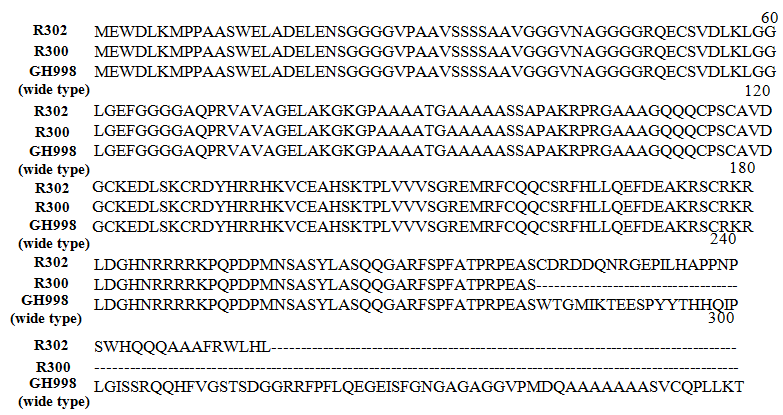


**C**

**D**

**E**

**
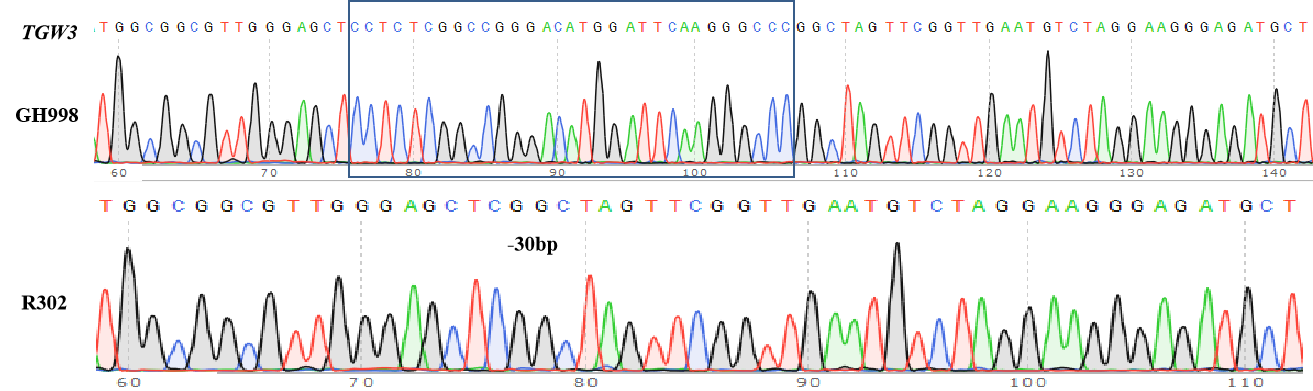
**


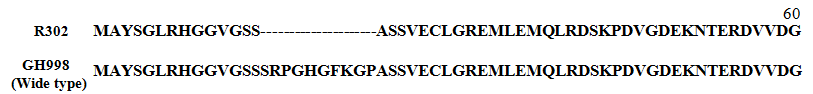


**Fig. S4 Sequencing and amino acid analyses of the two homozygous edited transgenic lines**

Note: **A** Sequencing analysis of the mutant line R302 and the control of GH998 at *GW8*mutation site.The blue box indicates the mutated base. **B** Sequencing analysis of the mutant line R300 and the control of GH998 at *GW8*mutation site. **C** *GW8* amino acid alignment of two mutants and the control GH998. **D** Sequencing analysis of the mutant line R302 and the control of GH998 at *TGW3* mutation site. **E** *TGW3* amino acid alignment of mutant line R302 and the control GH998.


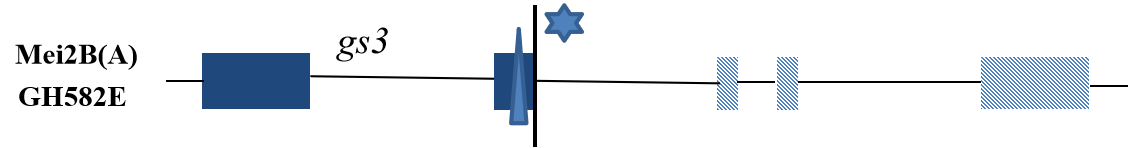


**A**


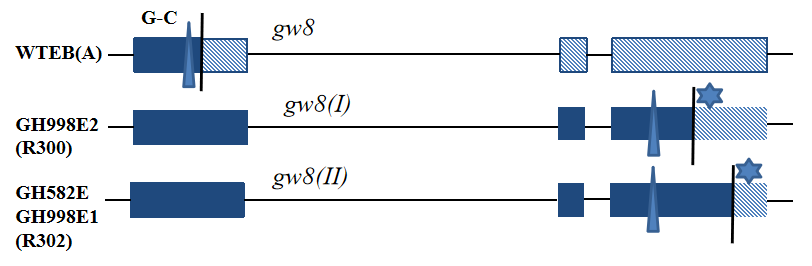


**B**

**C**


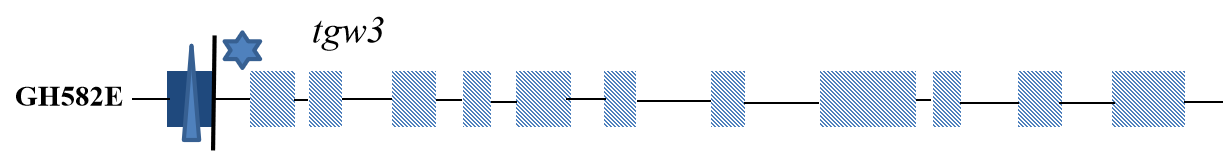


**Fig. S5 Schematic diagram of grain size gene mutations in parental lines**

Note: **A** Schematic diagram of *GS3* gene mutations. **B** Schematic diagram of *GW8* gene mutations. **C** Schematic diagram of *TGW3* gene mutation(Huang et al., 2022; Huang et al., 2023). The solid blue boxes represent the exons, hatched boxes represent 3’UTR, and the thin lines between the boxes represent the introns. Blue triangles represent base insertion and stop codons are marked by stars.

**Table S1 Primers used in this study**

| Primer name | Sequence (5'-3') |
| --- | --- |
| GS3-F2 | acagtacttgctgtctagcttt |
| GS3-R2 | actcccaacgttcagaaattaaatg |
| GW8-F | atgcgagtggtgatctgatctctgc |
| GW8-R | ctggaggtcgaccgagcact |
| GW8-F2 | cagctatgactctgttctttca |
| GW8-R2 | caggactgtgttcagctg |
| TGW3-F1 | agtgatgctggaggaaacgg |
| TGW3-R1 | tgcttttctagggtggacaaca |
| TGW3-F2 | gccactgtgcctttctgaga |
| TGW3-R2 | cctgtttctcgacatttggcc |
| TGW3-F3 | cggaactggttcttttgggg |
| TGW3-R3 | gcccccttgttcctcgaaaa |
| TGW3-F4 | gcacatcattttggccggta |
| TGW3-F4 | tgcaactccttcatgtaccaa |
| TGW3-F5 | tacaggctgtgtcatggcag |
| TGW3-R5 | gacattgctcacacacgcag |
| TGW3-F6 | gctgatggaagagcactttgg |
| TGW3-R6 | ctttgacctgcacattcggc |
| TGW3-F7 | ggcacaaggtattctgccca |
| TGW3-R7 | ggacgcccattaggtagacg |
| TGW3-F8 | cgtctacctaatgggcgtcc |
| TGW3-R8 | ccacactgagatgggcacat |
| TGW3-F | gcaagcaatggagtgaatactg |
| TGW3-R | agtgacagactgttccattacc |
| GW8-A1+ | cagtggtctcatgcaagctggagaacagcggcggc |
| GW8-A1- | cagtggtctcaaaacgccgccgctgttctccagct |
| GW8-A2+ | cagtggtctcatgcaccggcggcgagctgggagct |
| GW8-A2- | cagtggtctcaaaacagctcccagctcgccgccgg |
| GW8-B1+ | cagtggtctcatgcaagaccggaggcaagctggac |
| GW8-B1- | cagtggtctcaaaacgtccagcttgcctccggtct |
| GW8-B2+ | cagtggtctcatgcagtcagctccggcgaactcca |
| GW8-B2- | cagtggtctcaaaacgtccagcttgcctccggtct |
| TGW3-S1+ | cagtggtctcatgcaataatagctactacgatccg |
| TGW3-S1- | cagtggtctcaaaaccggatcgtagtagctattat |
| TGW3-S2+ | cagtggtctcatgcagaatccatgtcccggccgag |
| TGW3-S2- | cagtggtctcaaaacctcggccgggacatggattc |
| Hyg-F | acgtctgtcgagaagtttctgatc |
| Hyg-R | agtcaatgaccgctgttatgc |

**Table S2 Genotypes of rice quality-related genes in hybrid parents and hybrid combinations**

| Variety | Genotype of *GS3* | Genotype of *GW8* | Genotype of T*GW3* | Genotype of *Wx* | Genotype of *ALK* | Genotype of *Chalk5* |
| --- | --- | --- | --- | --- | --- | --- |
| Mei1B | *GS3* | *gw8* | *TGW3* | *Wx^b^* | *ALK* | *Chalk5* |
| Mei2B | *gs3* | *gw8* | *TGW3* | *Wx^b^* | *ALK* | *Chalk5* |
| WTB | *gs3* | *GW8* | *TGW3* | *Wx^b^* | *ALK* | *chalk5* |
| WTEB | *gs3* | *gw8* | *TGW3* | *Wx^b^* | *ALK* | *chalk5* |
| GH998 | *gs3* | *GW8* | *TGW3* | *Wx^b^* | *ALK* | *chalk5* |
| GH998E1 | *gs3* | *gw8(II)* | *TGW3* | *Wx^b^* | *ALK* | *chalk5* |
| GH998E2 | *gs3* | *gw8(I)* | *TGW3* | *Wx^b^* | *ALK* | *chalk5* |
| GH582 | *GS3* | *GW8* | *TGW3* | *Wx^b^* | *alk* | *Chalk5* |
| GH582E | *gs3* | *gw8(II)* | *tgw3* | *Wx^b^* | *alk* | *Chalk5* |
| Mei1A/GH998 | *GS3*/*gs3* | *gw8/GW8* | *TGW3/TGW3* | *Wx^b^* | *ALK* | *Chalk5*/*chalk5* |
| Mei2A/GH998 | *gs3/gs3* | *gw8/GW8* | *TGW3/TGW3* | *Wx^b^* | *ALK* | *Chalk5*/*chalk5* |
| Mei1A/GH998E2 | *gs3/gs3* | *gw8/gw8(I)* | *TGW3/TGW3* | *Wx^b^* | *ALK* | *Chalk5*/*chalk5* |
| Mei2A/GH998E2 | *gs3/gs3* | *gw8/gw8(I)* | *TGW3/TGW3* | *Wx^b^* | *ALK* | *Chalk5*/*chalk5* |
| Mei1A/GH582 | *GS3/GS3* | *gw8/GW8* | *TGW3/TGW3* | *Wx^b^* | *ALK/alk* | *Chalk5/Chalk5* |
| Mei2A/GH582 | *gs3/GS3* | *gw8/GW8* | *TGW3/TGW3* | *Wx^b^* | *ALK/alk* | *Chalk5/Chalk5* |
| Mei1A/GH582E | *GS3/gs3* | *gw8/gw8(II)* | *TGW3/tgw3* | *Wx^b^* | *ALK/alk* | *Chalk5/Chalk5* |
| Mei2A/GH582E | *gs3/gs3* | *gw8/gw8(II)* | *TGW3/tgw3* | *Wx^b^* | *ALK/alk* | *Chalk5/Chalk5* |
| WTA/GH998 | *gs3/gs3* | *GW8/GW8* | *TGW3/TGW3* | *Wx^b^* | *ALK* | *chalk5*/*chalk5* |
| WTEA/GH998 | *gs3/gs3* | *gw8/GW8* | *TGW3/TGW3* | *Wx^b^* | *ALK* | *chalk5*/*chalk5* |
| WTA/GH998E1 | *gs3/gs3* | *GW8/gw8(II)* | *TGW3/TGW3* | *Wx^b^* | *ALK* | *chalk5*/*chalk5* |
| WTEA/GH998E1 | *gs3/gs3* | *gw8/gw8(II)* | *TGW3/TGW3* | *Wx^b^* | *ALK* | *chalk5*/*chalk5* |
| WTA/GH998E2 | *gs3/gs3* | *GW8/gw8(I)* | *TGW3/TGW3* | *Wx^b^* | *ALK* | *chalk5*/*chalk5* |
| WTEA/GH998E2 | *gs3/gs3* | *gw8/gw8(I)* | *TGW3/TGW3* | *Wx^b^* | *ALK* | *chalk5*/*chalk5* |
| WTA/GH582 | *gs3/GS3* | *GW8/GW8* | *TGW3/TGW3* | *Wx^b^* | *ALK/alk* | *chalk5/Chalk5* |
| WTEA/GH582 | *gs3/GS3* | *gw8/GW8* | *TGW3/TGW3* | *Wx^b^* | *ALK/alk* | *chalk5/Chalk5* |
| WTA/GH582E | *gs3/gs3* | *GW8/gw8(II)* | *TGW3/tgw3* | *Wx^b^* | *ALK/alk* | *chalk5/Chalk5* |
| WTEA/GH582E | *gs3/gs3* | *gw8/gw8(II)* | *TGW3/tgw3* | *Wx^b^* | *ALK/alk* | *chalk5/Chalk5* |

Note: *GS3*-short grain, *gs3*-long grain; *GW8*-wide grain, *gw8*-slender grain; T*GW3*-short grain, *tgw3*-long grain; *alk*-high alkali spreading value, *ALK*-low Alkali spreading value; *Wx^b^* -low amylose content; *Chalk5*-hight Chalkiness, *chalk5*-low Chalkiness

**Table S3 Statistics of parents agronomic traits**

| variety | Grain length  (mm) | Gain width  (mm) | Ratio of grain length to width | Panicle length  (cm) | Grain number per panicle | Filled grain number per panicle | Seed-setting rate (%) | 1000 grain weight (g) | Effective tiller number | Plant height  (cm) | Grain weight per plant(g) |
| --- | --- | --- | --- | --- | --- | --- | --- | --- | --- | --- | --- |
| Mei1B(Mei1A) | 9.40±0.37 | 2.42±0.11 | 3.89±0.20 | 25.6±2.0 | 163.0±44.6 | 141.8±49.8 | 84.5±12.8 | 18.0±0.6 | 10.5±1.7 | 111.9±4.3 | 19.5±2.6 |
| Mei2B(Mei2A) | 10.14±0.40* | 2.42±0.09 | 4.19±0.25* | 27.7±2.0* | 204.5±34.3* | 148.4±26.0 | 73.0±8.8* | 19.2±0.4* | 11.0±1.0 | 112.6±2.9 | 22.4±3.0* |
| WTB(WTA) | 10.26±0.41 | 2.66±0.12 | 3.85±0.21 | 23.2±1.3 | 264.0±48.7 | 170.7±43.4 | 64.0±6.6 | 24.2±1.2 | 9.8±2.9 | 116.2±2.7 | 31.6±5.6 |
| WTEB(WTEA) | 10.63±0.42 | 2.40±0.16* | 4.41±0.25* | 24.9±1.2 | 285.2±36.0 | 187.6±31.4 | 65.9±8.1 | 23.0±1.8 | 11.7±2.5 | 112.9±2.2 | 32.3±7.6 |
| GH582 | 8.61±0.24 | 2.82±0.10 | 3.05±0.13 | 28.0±1.7 | 199.8±35.1 | 158.2±31.6 | 79.0±4.0 | 21.0±0.2 | 9.2±1.1 | 100.9±6.8 | 24.5±4.9 |
| GH52E | 10.20±0.42* | 2.46±0.12* | 4.16±0.23* | 27.0±2.1 | 184.2±36.4 | 137.8±24.2 | 75.7±11.8 | 20.6±1.1 | 10.8±1.5 | 99.6±2.8 | 22.5±4.5 |
| GH998 | 9.59±0.36 | 2.65±0.13 | 3.63±0.25 | 22.9±1.5 | 161.6±11.7 | 129.8±12.2 | 80.5±8.6 | 21.7±0.5 | 8.6±0.9 | 104.0±2.8 | 26.9±3.0 |
| GH998E1 | 9.99±0.53 | 2.31±0.10* | 4.34±0.35* | 23.3±1.4 | 130.8±42.1 | 113.2±47.6 | 84.5±12.9 | 17.4±0.3 | 11.0±0.7 | 104.2±1.2 | 26.2±3.9 |
| GH998E2 | 10.75±0.29* | 2.23±0.24* | 4.87±0.52* | 23.3±1.5 | 138.8±43.8 | 108.0±47.4 | 84.2±0.1 | 18.4±0.01 | 11.2±1.3 | 106.2±1.2 | 25.5±3.5 |

Note: All data are given as means ±s.e.m. * indicated that the edited lines is significant difference at P levels 0.001 comparing to unedited lines (n=10 for grain length, grain width and ratio of grain length to width and n=5 for the other traits). The data of Mei1B(Mei1A) and Mei2B(Mei2A) were cited From Huang et al. 2022, and the data of GH582 and GH582E were cited from Huang et al. 2023.

**Table. S4 Grouping and corresponding genotypes of hybrid combinations**

| Group | Hybrid combination | | Genotype |
| --- | --- | --- | --- |
| **Ⅰ** | **Ⅰ-1** | Mei1A/GH998 | *GS3/gs3****-****GW8/gw8* |
|  | **Ⅰ-2** | Mei2A/GH998 | *gs3/gs3****-****GW8/gw8* |
|  | **Ⅰ-3** | Mei1A/GH998E2 | *GS3/gs3****-****gw8/gw8(I)* |
|  | **Ⅰ-4** | Mei2A/GH998E2 | *gs3/gs3****-****gw8/gw8(I)* |
| **Ⅱ** | **Ⅱ-1** | Mei1A/GH582 | *GS3/GS3****-****gw8/GW8****-****TGW3/TGW3* |
|  | **Ⅱ-2** | Mei2A/GH582 | *gs3/GS3****-****gw8/GW8****-****TGW3/TGW3* |
|  | **Ⅱ-3** | Mei1A/GH582E | *gs3/GS3****-****gw8/gw8(II)****-****TGW3/tgw3* |
|  | **Ⅱ-4** | Mei2A/GH582E | *gs3/gs3****-****gw8/gw8(II)****-****TGW3/tgw3* |
| **Ⅲ** | **Ⅲ-1** | WTA/GH998 | *gs3/gs3-GW8/GW8* |
|  | **Ⅲ-2** | WTEA/GH998 | *gs3/gs3-gw8/GW8* |
|  | **Ⅲ-3** | WTA/GH998E1 | *gs3/gs3-GW8/gw8(II)* |
|  | **Ⅲ-4** | WTEA/GH998E1 | *gs3/gs3-gw8/gw8(II)* |
|  | **Ⅲ-5** | WTA/GH998E2 | *gs3/gs3-GW8/gw8(I)* |
|  | **Ⅲ-6** | WTEA/GH998E2 | *gs3/gs3-gw8/gw8(I)* |
| **Ⅳ** | **Ⅳ-1** | WTA/GH582 | *gs3/GS3-GW8/GW8-TGW3/TGW3* |
|  | **Ⅳ-2** | WTEA/GH582 | *gs3/GS3-gw8/GW8-TGW3/TGW3* |
|  | **Ⅳ-3** | WTA/GH582E | *gs3/gs3-GW8/gw8(II)-TGW3/tgw3* |
|  | **Ⅳ-4** | WTEA/GH582E | *gs3/gs3-gw8/gw8(II)-TGW3/tgw3* |

**Table S5 Primers of fluorescent marker**

| Primer name | Sequence (5'-3') | Fluorescence value and corresponding genotype |
| --- | --- | --- |
| Chalk5a-FT | gaaggtcggagtcaacggattcagacggtgccgtttgtagt | HEX Value: Low chalkiness |
| Chalk5a-FC | gaaggtgaccaagttcatgctcagacggtgccgtttgttgc | FAM Value: High chalkiness |
| Chalk5a-R1 | tttgtcactgtcttctatctcacc | - |
| alk-Ftt | gaaggtgaccaagttcatgcttacaaggagagctggaggggtt | FAM Value: Low Alkali spreading value |
| alk-Fgc | gaaggtcggagtcaacggatttacaaggagagctggagggggc | HEX Value: How alkali spreading value |
| alk-R | ctgaggtcctgcgacatgc | - |
| RWx-Fg | gaaggtgaccaagttcatgcttcatcaggaagaacatctgcaagg | FAM Value: High amylose content |
| RWx-Ft | gaaggtcggagtcaacggatttcatcaggaagaacatctgcaagt | HEX Value: Low amylose content |
| RWx-R | ggaaaaacgagcaatgaaagatgc | - |
| GW8-Ra | gaaggtgaccaagttcatgctcagagatgagaggctgcgca | FAM Value: slender grain |
| GW8-Rc | gaaggtcggagtcaacggatt cagagatgagaggctgcgcc | HEX Value: wider grain |
| GW8-F | tccagcccaccgagcacat | - |
